# Surface localized magnetism in transition metal doped alumina

**DOI:** 10.1038/s41598-021-85791-5

**Published:** 2021-03-19

**Authors:** Erik C. Nykwest, Dennis Trujillo, S. Pamir Alpay

**Affiliations:** 1 National Security Sciences: Nuclear Nonproliferation Division, Oak Ridge National Laboratory, Oak Ridge, TN 37831 USA; 2 Department of Materials Science and Engineering, University of Connecticut, Storrs, CT 06269 USA; 3 Department of Physics, University of Connecticut, Storrs, CT 06269 USA

**Keywords:** Condensed-matter physics, Magnetic properties and materials, Spintronics, Surfaces, interfaces and thin films, Theory and computation, Atomistic models, Electronic structure, Materials science, Materials for energy and catalysis

## Abstract

Alumina is a structural ceramic that finds many uses in a broad range of applications. It is widely employed in the aerospace and biomedical sectors due to its stability at high temperatures and in harsh chemical environments. Here, we show that magnetism can be induced at alumina surfaces by doping with 3d transition metals. We analyze the electronic structure, spin magnetic moments, and spin density of $\alpha$-Al$_{2}$O$_{3}$ as a function of both dopant species (Sc, Ti, V, Cr, Mn, Fe, Co, Ni, Cu) and depth using first principles calculations. Our results show that all dopants, with the exception of Sc, produce magnetic moments that are concentrated to the surface of alumina with varying degrees of delocalization. It is seen that all of the dopants are at least meta-stable on the surface and must overcome an energy barrier of 0.19–1.14 eV in order to diffuse from the surface into the bulk. As a result of judiciously doping with select 3*d* transition metals the surface of alumina can be made magnetic. This could lead to novel applications in data storage, catalysis, and biomedical engineering through an added surface functionality.

Magnetic semiconducting materials exhibit a large range of electronic and magnetic functionalities which have been exploited in fields as diverse as data storage^1^, catalysis^2–5^, photovoltaics^6^, and medicine^7^. Aluminum oxide (Al$_{2}$O$_{3}$), in particular, is an extremely technologically relevant material and has been studied extensively due to its low thermal conductivity^8^, high chemical stability^9^, lasing ability^10^, and relative low degree of lattice mismatch relative to a large number of semiconducting insulators^11^. The further enhancement of the electronic and magnetic properties of Al$_{2}$O$_{3}$ is of great interest where doping and geometric alterations (strain, surface enhancement) are the most promising means of improving the material response.

It has been demonstrated that substituting a transition metal dopant could produce alumina that possesses both unpaired electrons and a net magnetic moment^12^. In addition, it was also determined that the delocalization of these unpaired electrons varied greatly between dopants^13^, which may affect the final magnetic properties of the doped system^14^. Previous work in metallic systems^15–17^ has pointed toward enhanced magnetism near surfaces, where a transition in spin density from the bulk to the surface region was observed. Likewise, the presence of spin density waves (SDW) have been evaluated via *ab initio* simulations and experiments for multiple metal^18–22^ and metal oxide materials^23–26^. Changes in the magnetic order have been observed in doped topological insulators^27,28^, indicating a bulk-surface transition in the spin density wave for insulating materials. Considering alumina is a wide bandgap ceramic which has been used in many applications, an observed surface localized magnetization and change in magnetic behavior has implications for developing novel magnetic data storage devices, photovoltaics and catalysis.

This work focuses on demonstrating surface localized magnetic behavior of 3*d* transition metal (Sc, Ti, V, Cr, Mn, Fe, Co, Ni, Cu) doped alumina. These elements were chosen due to their exhibited magnetic properties (Fe, Co, Ni, Cr, Mn) and potential to alter the electronic behavior of alumina based on previous dopant effect studies^29–31^. The (0001) surface of $\alpha$-alumina, terminated with a single atomic layer of Al, is both the most stable (lowest energy) and most commonly studied surface^32–35^. Along the $\langle$0001$\rangle$ direction of the $\alpha$-alumina unit cell, each layer is comprised either of a single Al atom, or three O atoms. These layers can be grouped into stoichiometric balanced trilayers containing 2 Al atoms and 3 O atoms. From these trilayers $\alpha$-alumina slabs of arbitrary thickness can be constructed. Density functional theory was used to determine the magnetic behavior of transition metal doped $\alpha$-alumina slabs as a function of dopant depth from the surface. The preference for a dopant to be placed at the surface or in the bulk was determined via defect formation energy calculations. The total magnetic moment, and the degree of its delocalization from the dopant atom is reported in both a visual and numerical format.

After extensive convergence testing it was observed that a slab five trilayers thick (fifteen atomic layers) could accurately represent both the bulk and the (0001) surface of $\alpha$-alumina. This five trilayer slab unit cell is shown in Fig. 1. A $2 \times 2 \times 1$ super cell of these slab unit cells with periodic boundary conditions was utilized for all calculations with a 20 Å vacuum gap introduced along the $\langle$0001$\rangle$ direction to prevent interactions between periodic images. While five trilayers are thicker than most previous research regarding $\alpha$-alumina, those studies have focused on examining only the surface of alumina (e.g. surface energy and adatoms^32–35^). This work, however, is concerned with the tendency of dopants to segregate either towards the surface or into the bulk, and as such must represent both the bulk and surface accurately.

**Figure 1:**
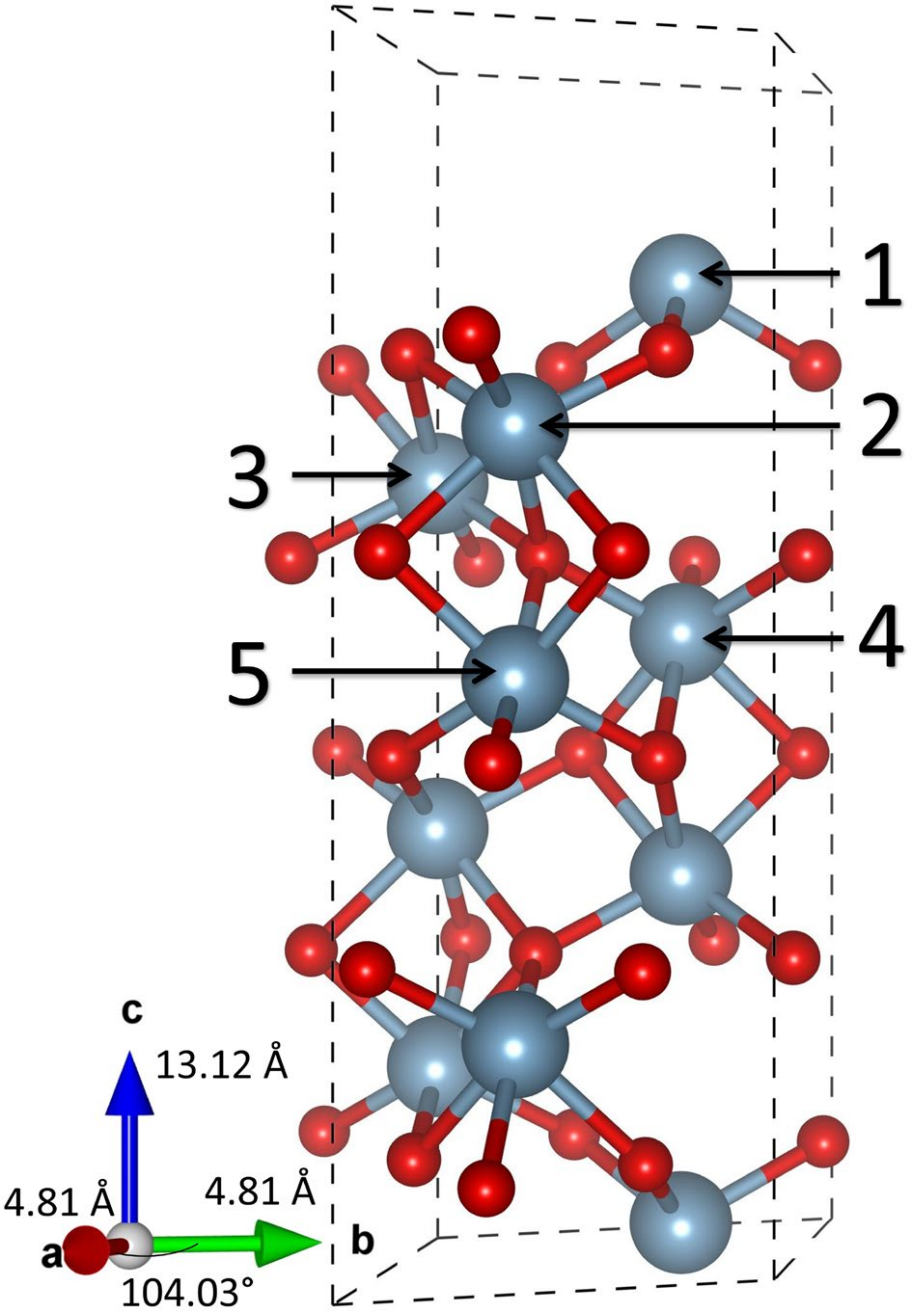
Five trilayer slab unit cell of $\upalpha$-alumina. The larger (blue) atoms are Al and the smaller (red) are O. The unique substitutional Al doping sites have been labeled 1–5 where 1 is surface like and 5 is bulk like.

Density functional theory (DFT) was utilized to determine the defect formation energy and the magnetic behavior of transition metal doped $\alpha$-alumina in the R$\bar{3}$c trigonal crystal system where a (0001) orientated slab model was considered. Many factors were taken into consideration during convergence testing: slab thickness, vacuum spacing, surface energy, interlayer spacing, the bond lengths of the bulk-like environment aluminum atoms, and the total energy of the un-doped slab. In particular, the surface energy was converged to better than 0.002 J/m$^{2}$, and the interlayer spacing and bulk-like environment aluminum bond lengths better than 1%. The settings used in these calculations are similar to the ones that were rigorously tested and used in our previous studies^12,13^ which involved an examination of bulk alumina doped with transition metals (Sc-Cu). First principles studies were performed within the general gradient approximation^36^ (GGA) with Perdew–Burke–Ernzerhof^37^, (PBE) parameterization, utilizing the projector-augmented plane wave (PAW) pseudopotentials, as implemented in the Vienna Ab Initio Simulation Package (VASP)^38–40^, using 0.005 Gaussian smearing. The atomic positions of each doped alumina super cell were relaxed simultaneously via damped molecular dynamics with a plane wave cut off energy of 500 eV and a $2 \times 2 \times 1$ gamma centered Monkhorst–Pack K-mesh until the forces on each atom were less than $1 \times 10^{-6}$ eV/Å. After the super cell was relaxed the wave functions and charge density were recalculated with a plane wave cut off of 800 eV without further relaxation. It was seen previously that the total energy is converged to less than 6 meV/atom at a cutoff of 500 eV and less than 0.1 meV/atom at a cutoff of 800 eV. The ground state energy for isolated atoms were calculated using a 800 eV cut off with the gamma point only along with a Gaussian smearing of 0.001 through an asymmetric simulation cell of dimension $20\times 21\times 22$ Å$^{3}$.

To evaluate the tendency of each dopant to segregate either towards the surface or into the bulk the Defect Formation Energy (DFE) was calculated for each dopant, at each unique Al substitutional doping site (labeled 1–5 in Fig. 1), using Eq. (1) below. The energetically preferred doping depth may then be determined by identifying the doping site with the minimum defect formation energy.

The defect formation energy may also be used to check the convergence of the model system as DFE of the deepest bulk-like layer in the slab should equal to that of the periodic bulk system, if the slab is well converged. We define the DFE as:

1 $$\begin{aligned} {DFE = (E_{Doped} + E_{Al}) - (E_{Pure} + E_{TM})} \end{aligned}$$

where E$_{\text {Doped}}$ is the total energy of the doped alumina, E$_{\text {Al}}$ is the energy of an isolated aluminum atom, E$_{\text {Pure}}$ is the energy of undoped bulk alumina, and E$_{\text {TM}}$ is the energy of the isolated dopant atom. While this work strives to report accurate defect formation energies, it is important to note that the absolute value of the defect formation energy (which is a function of slab thickness) is not as important as the change in the defect formation energy between doping sites. The DFE values for each respective dopant are listed in Table 1. Accordingly, the difference in the defect formation energy relative to the deepest bulk-like doping site (RDFE) is also calculated via,

**Table 1:** Defect formation energy for the considered dopants at positions 1–5 in the slab (see Fig. 1).

DFE (eV)	Sc	Ti	V	Cr	Mn	Fe	Co	Ni	Cu
Bulk	− 1.19	− 0.41	0.70	2.17	3.62	3.82	3.66	4.94	7.06
Bulk-Like	− 1.39	− 0.58	0.58	2.09	3.45	3.79	3.64	4.86	6.95
Difference	0.20	0.17	0.13	0.08	0.17	0.03	0.01	0.08	0.11

2 $$\begin{aligned} {RDFE(i) = DFE(i)-DFE(5)} \end{aligned}$$

where RDFE(*i*) is the relative increase/decrease in the defect formation energy DFE(*i*) when a dopant is placed in doping site *i* instead of the bulk-like doping site *i*=5.

The defect formation energy was calculated for each dopant at each unique Al substitutional doping site. The defect formation energy as a function of the dopant is shown in Fig. 2. The trend is in agreement with our past work on bulk doped alumina^13^, with scandium and titanium being the most energetically favorable dopants introduced to the system overall, and nickel and copper the least favorable dopants for each respective aluminum substitutional site. This can be explained by the preference of Sc ([Ar]3d$_{1}$4s$_{2}$) and Ti ([Ar]3d$_{2}$4s$_{2}$) to donate 3 electrons to the octahedrally coordinated oxygen atoms, bringing their valency closer to Ar than the other considered dopants. More details on the trends of defect formation energy can be found in^13^.

While the defect formation energy allows us to make inferences about relative difficultly of embedding each dopant atom into an alumina slab, the relative defect formation energies may be used to draw conclusions about the stability of the doped systems once the atom is already embedded. It can be seen from Fig. 3 that most of the dopants have a global minimum RDFE at position 1 and thus prefer placement at the surface, with the exception of chromium which prefers to be inside the slab and away from the surface. Similarly, most of the dopants exhibit a global maximum RDFE at position 2, with the notable exceptions of iron and cobalt which do not possess an energy barrier. In order for an atom to migrate from the surface (position 1) to the “bulk” (position 5), or vice versa, it would have to overcome this energy barrier. Accordingly, the global maximum RDFE is reported as an energy barrier in Table 2.

When considered in tandem, the segregation energy and the energy barrier can be used to categorize the long-term stability of the doped systems against diffusion into the bulk. The total energy required for a dopant to segregate from the surface into the bulk is the sum of the segregation energy and this energy barrier. The larger this sum, the more difficult it is for a dopant to segregate into the bulk. For example, of the dopants examined, Sc is the most robust element against diffusion into the bulk. In order for a Sc atom to migrate from the surface into the bulk, it would require an energy of 1.14 eV. On the other end of the spectrum, Co is the least resistant against diffusion and would only require 0.19 eV of energy to migrate into the bulk. The dopants from highest to lowest predicted long-term stability are: Sc, Ti, Fe, Cu, Mn, V, Ni, Cr, Co.

**Figure 2:**
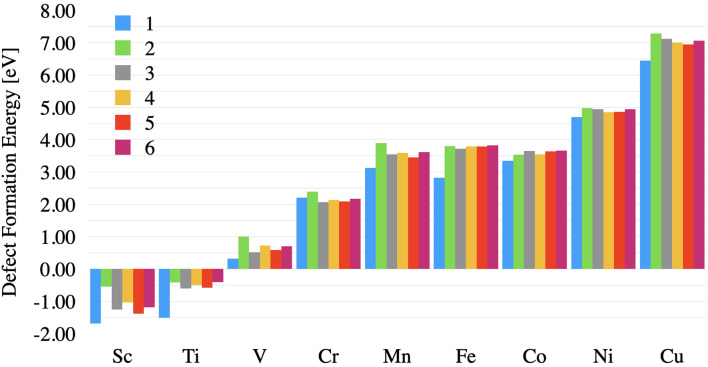
Defect formation energy of transition metal doped alumina as a function of dopant species and surface depth (1–5 as illustrated in Fig. 1). Position 6 shows the defect formation energy of bulk doped $\upalpha$-alumina reported in references^12,13^.

**Figure 3:**
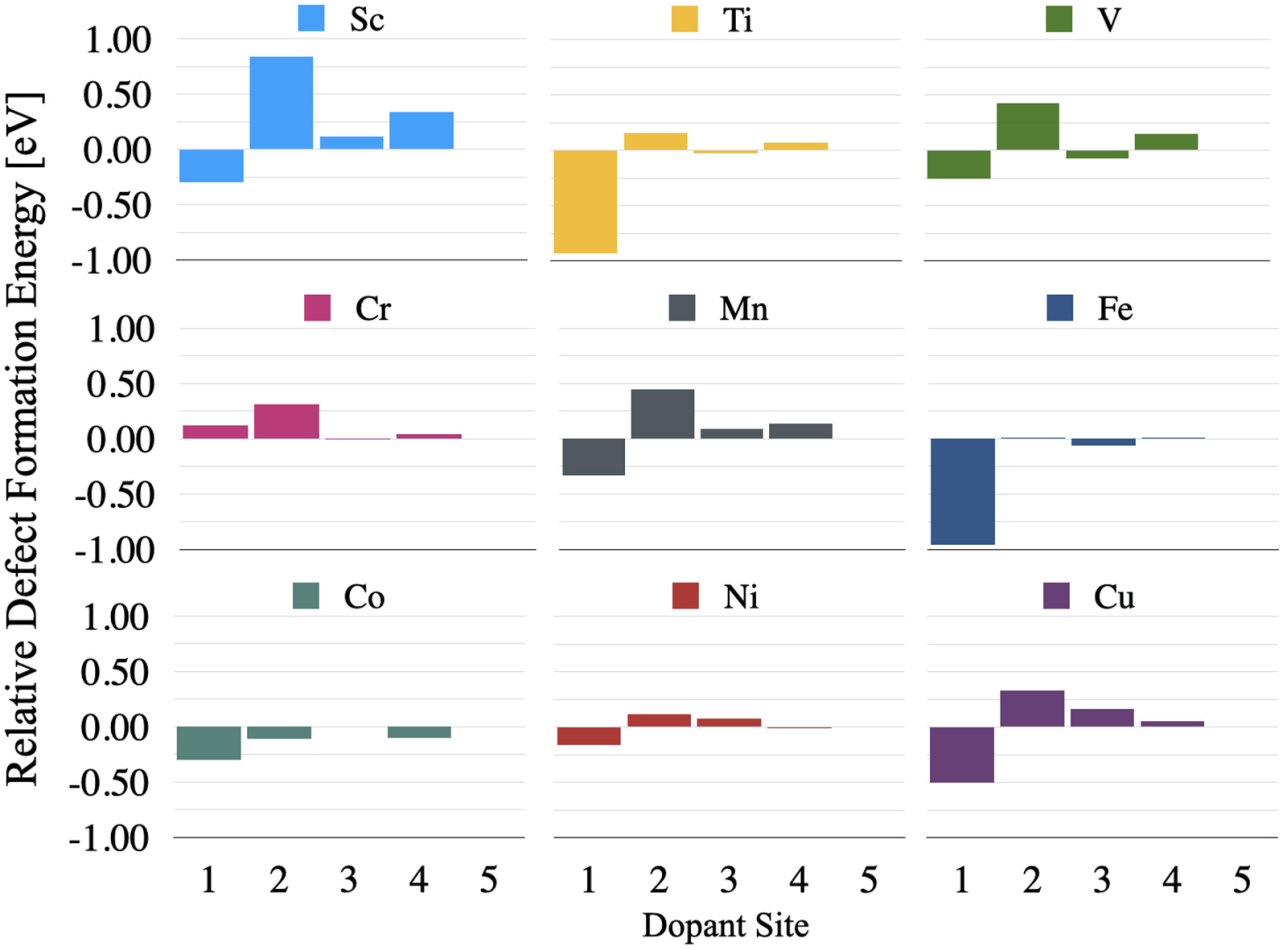
Defect formation energy, in eV, relative to bulk-like doping (position 5) for each respective dopant. Negative energies suggest the doping site is more stable than bulk-like doping, while positive energies suggest the doping site is less stable than bulk-like doping.

**Table 2:** Relative defect formation energy for the considered dopants at positions 1–5 in the slab (see Fig. 1).

RDFE (eV)	Sc	Ti	V	Cr	Mn	Fe	Co	Ni	Cu
1 (Surface)	− 0.30	− 0.93	− 0.26	0.12	− 0.33	− 0.96	− 0.30	− 0.16	− 0.50
2	0.84	0.16	0.43	0.31	0.45	0.01	− 0.11	0.12	0.33
3	0.12	− 0.03	− 0.07	− 0.01	0.09	− 0.06	0.00	0.08	0.16
4	0.34	0.07	0.15	0.04	0.14	0.01	− 0.10	− 0.01	0.05
5 (Bulk-Like)	0.00	0.00	0.00	0.00	0.00	0.00	0.00	0.00	0.00
Segregation Energy (eV)	− 0.30	− 0.93	− 0.26	0.12	− 0.33	− 0.96	− 0.30	− 0.16	− 0.50
Energy Barrier (eV)	0.84	0.16	0.43	0.31	0.45	0.01	0.00	0.12	0.33

The magnetic and electronic properties of transition metal doped alumina, with the dopants placed at various distances from the surface, was also determined. Undoped alumina is diamagnetic and contains no unpaired electrons. Doping alumina with transition metal elements adds unpaired electrons into the system which induce a net magnetic moment. The presence of unpaired electrons in the system, and an asymmetric Density of States (DOS), signals that it is no longer diamagnetic. Our previous work found that some dopants induce a net magnetic moment that is localized near the dopant atom (e.g. iron), while other dopants (e.g. copper) introduce unpaired electrons that delocalize and induce a net magnetic moment on neighboring oxygen atoms^12,13^. This work finds an additional unexpected functional property of transition metal doped alumina. When doped into the top-most surface layer (position 1) of alumina, the induced magnetic moment is confined almost entirely to the surface.

The total magnetic moment in each doped slab as a function of doping site was calculated. For doping sites 2–5, both the total magnetic moment and the magnetic moment distribution (localized vs delocalized) are consistent with bulk calculations^12,13^. The total magnetic moment and the magnetic moment distribution for atoms doped into the surface layer (position 1) are quite different from bulk predictions due to a change in the coordination environment and are reported in Table 3. With regards to surface doping, the dopants exhibiting the highest total magnetic moment are iron and cobalt with a calculated value of 4.60 $\upmu$B and 3.73 $\upmu$B respectively. The dopants exhibiting the highest localized magnetic moment are iron and manganese with a reported value of 3.81 $\upmu$B and 3.31 $\upmu$B respectively. Finally, the dopants exhibiting the highest delocalized magnetic moment are copper and nickel with 1.01 $\upmu$B, cobalt with 0.94 $\upmu$B, and iron with 0.79 $\upmu$B.

The pronounced difference between the different dopants in magnetic delocalization and magnetic behavior at the surface is evident when you examine the spin density distribution. The spin density is the difference between the charge density for the spin up electrons and the spin down electrons and identifies regions of space where unpaired electrons are likely to be found. The spin density for manganese, iron, and copper doped alumina slabs in position 1 and 5 can be seen in Fig. 5a–c and d–f respectively. The spin densities for position 5 are in agreement with spin densities predicted by bulk calculations^12,13^.

The spin densities for most of the examined dopants can be placed into two broad categories, localized like Mn, or delocalized like Cu. A high degree of localization is observed where the spin density is concentrated on the dopant atom and the nearest neighboring oxygen atoms (Fig. 5a). Spin localization is preserved for Mn in the bulk configuration (Fig. 5d). The elements that generate similar spin densities to Mn, but with smaller total magnetic moments, are Ti, V, and Cr.

In contrast to manganese, the spin density generated by copper is widely delocalized across the surface of the alumina slab, but is still highly concentrated in the first few atomic layers (Fig. 5c). A large degree of delocalization is also observed for copper in bulk, consistent with its behavior at the surface. Surface localized magnetism is technologically relevant to developing novel magnetic storage media in addition to influencing catalytic reactions. The elements that generate similar spin densities to copper are nickel and cobalt.

Iron is a notable exception to these two groups. Fe represents a transition between the localized dopants (Ti, V, Cr, Mn) and the delocalized dopants (Co, Ni, Cu). Fe generates both the largest total magnetic moment of all the dopants when doped into position 1, which is represented by the large spin density distribution centered directly on the Fe atom (Fig. 5b). However, Fe also induces a delocalized magnetic moment of 0.8$\upmu _\text {B}$ that spreads across the surface and is concentrated in the first few atomic layers. In the bulk configuration the spin density is localized near the dopant atom (Fig. 5).

The differences between the bulk and surface spin densities are due to differences in the local coordination environment. In bulk doped $\upalpha$-alumina the transition metal d-states undergo octahedral crystal field splitting as they are octahedrally coordinated by six oxygen atoms^12^, causing the orbital loading to deviate from Hund’s rule. When doped into the surface of $\alpha$-alumina however, there are only three nearest neighboring oxygen atoms so the dopants are no longer octahedrally coordinated and the d-states undergo a different crystal field splitting. The surface crystal field splitting is not large enough to cause the orbital loading to deviate from Hund’s rule, which leads to differences between the surface doped spin density and the bulk doped spin density.

In bulk doped $\upalpha$-alumna it has been shown that the high energy $t_{2g}$ states are responsible for the delocalized spin density^13^. When these high energy $t_{2g}$ states are unoccupied, the spin density is localized to the dopant atom, but when they are occupied the spin density is delocalized onto the neighboring oxygen atoms. We present the element resolved density of states (DOS) in Fig. 4, illustrating the contribution of each atomic species to the total density of states for the surface doped configuration. Comparing the DOS of each doped system to its spin density, it can be seen that the mechanism of spin delocalization for surface doping is the same as in the bulk. When the two highest energy spin up transition metal d states (analogous to the $t_{2g}$ state for bulk doped $\upalpha$-alumina) are occupied, then the spin density delocalizes from the dopant atom onto the neighboring oxygen atoms.

Asymmetries in the DOS are observed between the spin up and down states for each of the dopant cases presented here, with the with the only symmetric DOS being scandium doped $\upalpha$-alumina. This is most visible in the existence of unpaired defect states in the band gap. The DOS for Cu doping is asymmetric, but it is harder to identify visually as most of the transition metal d states have hybridized with valence band. These unpaired spin states allow for the existence of stable ground state with a net spin and the emergence of magnetic behavior. We do not attempt to categorize these materials as ferromagnetic or anti-ferromagnetic as we only introduced one dopant atom into the simulation cell and thus can only produce ferromagnetic ordering, as all periodic images of this defect have the same spin. However, due to the presence of unpaired electrons in the system and the asymmetry of the DOS, we can state that the system is no longer diamagnetic.

**Figure 4:**
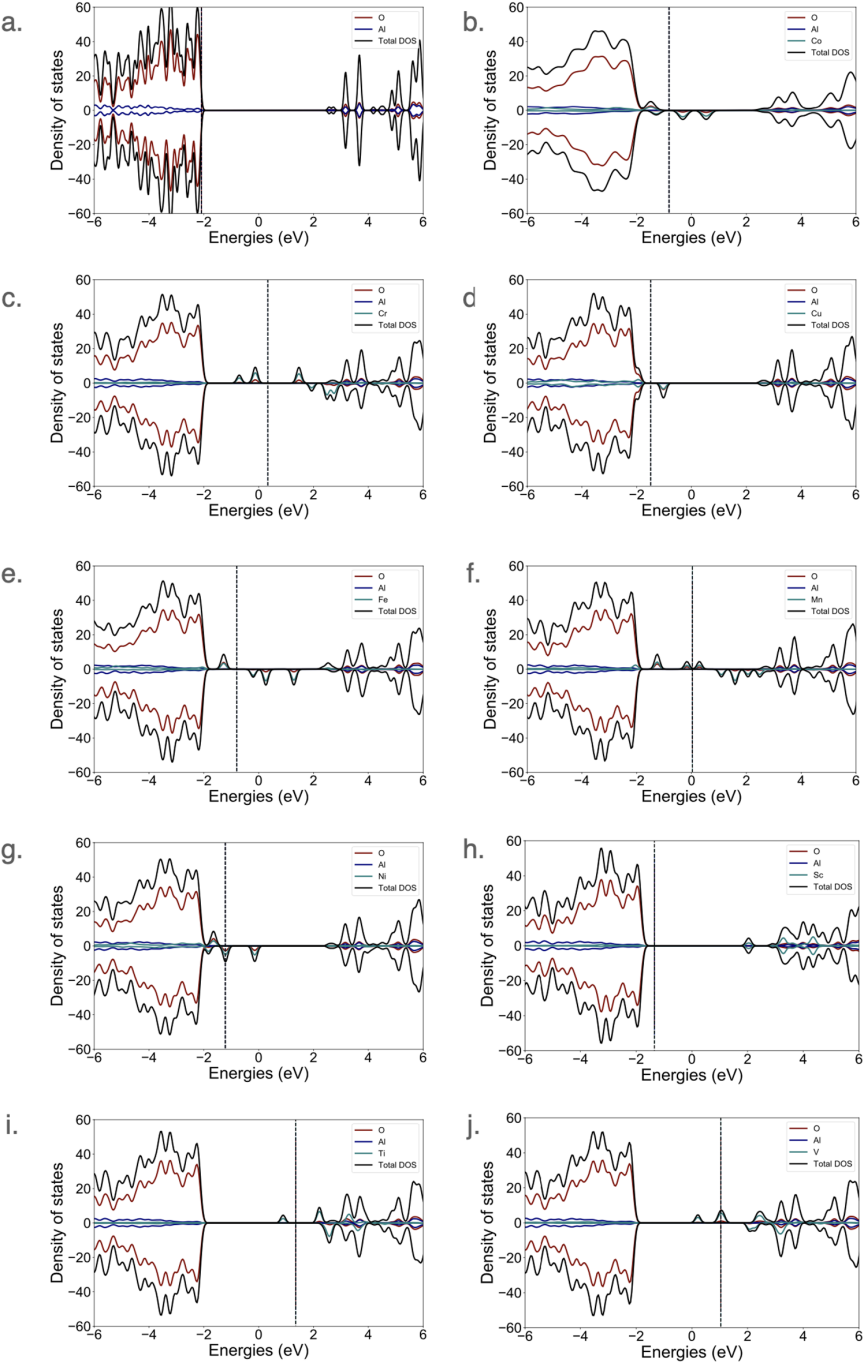
Element resolved density of states of pure and surface doped $\alpha$-alumina illustrating the introduction of defect states in the band gap and spin asymmetries for the doped configurations. The pristine case (**a**) is compared to Co (**b**), Cr (**c**), Cu (**d**), and Fe (**e**), Mn (**f**), Ni (**g**), Sc (**h**), Ti (**i**), and V (**j**) dopants in the surface dopant configuration illustrated as position 1 in Fig. 1. The fermi level is indicated by a vertical dashed line.

In conclusion, cross referencing the magnetic results with the previously discussed predicted stability against diffusion, the most promising dopants in our data set can determined. Iron was observed to exhibit strong preferential placement at the surface of alumina slabs compared to placement in the bulk. In addition, strong localization and a high total magnetic moment likewise make iron an ideal candidate for surface doping in alumina. Compared to iron, all of the other dopants either produce a smaller total magnetic moment or are less stable against migration from the surface into the interior of the slab.

For applications where delocalization of the magnetic moment is more important than the total magnetic moment, copper is a suitable alternative to iron. The delocalized portion of the magnetic moment induced by copper is one of the largest of the elements examined and it predicted to be significantly more resilient against migration into the bulk than cobalt or nickle. Given these considerations there is a high potential for utilizing iron and copper dopants in surface sensitive devices for potential applications in data storage or catalysis.

While this work was constrained to examining $\upalpha$-alumina, it is important to note that many of the metastable phases of alumina are characterized by high porosity and large surface-area to volume ratios^41,42^. In the future, it may be worthwhile to examine how surface doping changes the surface energy as a function of dopant concentration, as this may have a (de)stabilizing effect on the meta-stable phases relative to the $\upalpha$-phase. The methods presented in this manuscript would provide an excellent starting point for such future work regarding surface enhanced magnetism in alumina and other ceramics.

**Table 3:** The spacial distribution of the magnetic moment, in $\upmu \text {B}$, in the slab for each dopant species when doped into the surface (position 1 in Fig. 1). The portion of the magnetic moment attributed to the dopant atom is listed under Local, the sum of the magnetic moments on all atoms except the dopant is listed under Non-local, and the total magnetic moment induced in the slab (sum over all atoms) is listed under Total.

	Sc	Ti	V	Cr	Mn	Fe	Co	Ni	Cu
Local ($\upmu \text {B}$)	0.00	0.66	1.69	2.47	3.31	3.81	2.79	1.75	0.79
Non-local ($\upmu \text {B}$)	0.00	− 0.04	− 0.03	0.04	0.21	0.79	0.94	1.01	1.01
Total ($\upmu \text {B}$)	0.00	0.62	1.66	2.51	3.52	4.60	3.73	2.76	1.80

**Figure 5:**
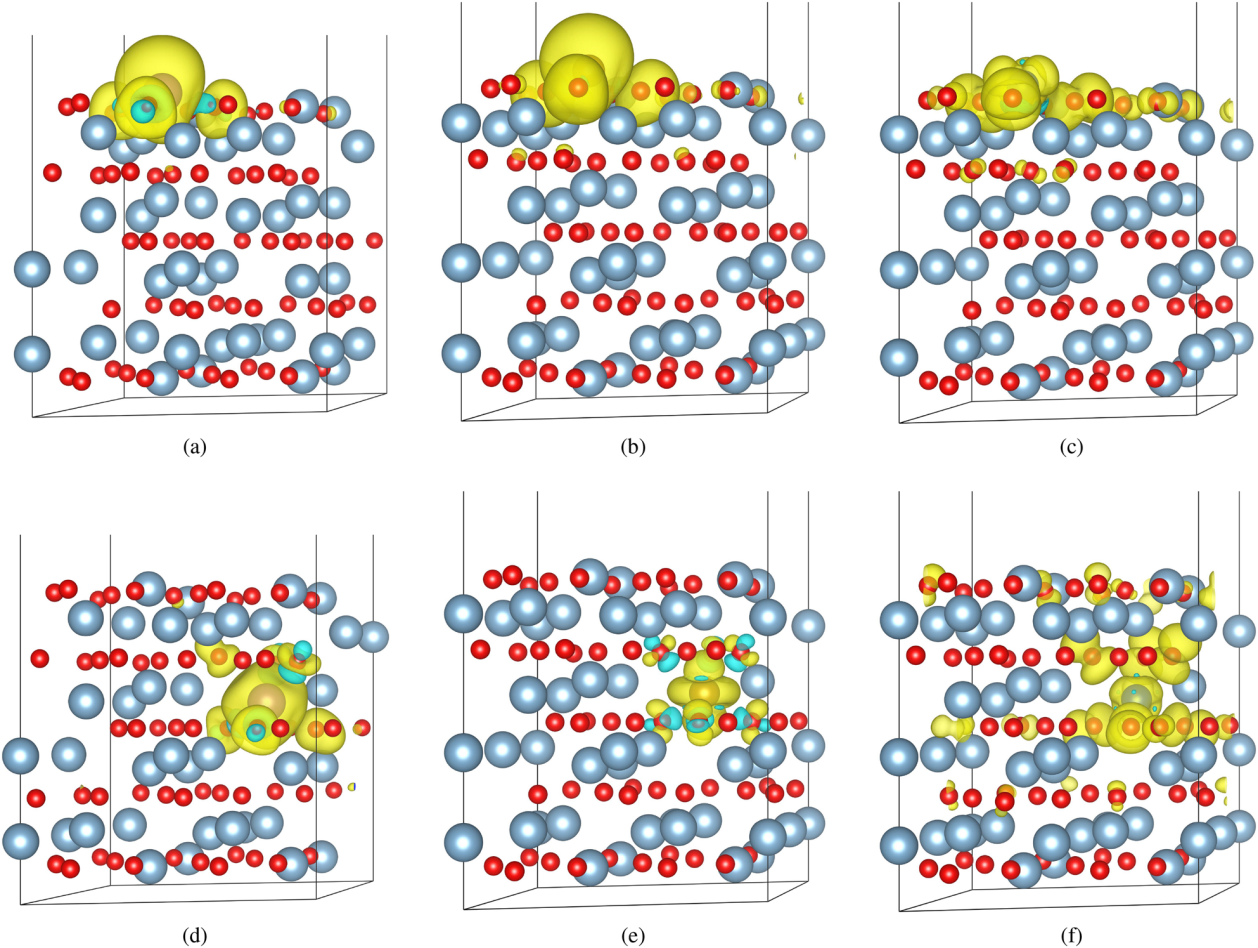
The spin density distribution 0.0015 $\upmu _\text {B}{\mathring{\rm A}}^-3$ isosurfaces for (**a**) Mn, (**b**) Fe, and (**c**) Cu doped into the surface of $\alpha$-alumina along with the spin density distribution for (**d**) Mn, (**e**) Fe, and (**f**) Cu doped into the bulk-like region of the slab. The larger (Blue) atoms are Al, the smaller (red) atoms are O, and the remaining atom is the dopant. Yellow represents the up (majority) spin isosurfaces, aquamarine represents down (minority) spin isosurfaces. Some Al and O atoms were removed from the super cell to increase visibility.
